# Hybrid Magnetic Lipid-Based Nanoparticles for Cancer Therapy

**DOI:** 10.3390/pharmaceutics15030751

**Published:** 2023-02-23

**Authors:** Marcela Tavares Luiz, Jessyca Aparecida Paes Dutra, Juliana Santos Rosa Viegas, Jennifer Thayanne Cavalcante de Araújo, Alberto Gomes Tavares Junior, Marlus Chorilli

**Affiliations:** 1 School of Pharmaceutical Sciences, São Paulo State University (UNESP), Araraquara 14800-903, SP, Brazil; 2 Instituto de Investigação e Inovação em Saúde, Universidade do Porto, 4200-135 Porto, Portugal

**Keywords:** magnetic hyperthermia, liposomes, nanocarriers, nanoemulsion, nanostructured lipid carrier, microemulsion, solid lipid nanoparticles

## Abstract

Cancer is one of the major public health problems worldwide. Despite the advances in cancer therapy, it remains a challenge due to the low specificity of treatment and the development of multidrug resistance mechanisms. To overcome these drawbacks, several drug delivery nanosystems have been investigated, among them, magnetic nanoparticles (MNP), especially superparamagnetic iron oxide nanoparticles (SPION), which have been applied for treating cancer. MNPs have the ability to be guided to the tumor microenvironment through an external applied magnetic field. Furthermore, in the presence of an alternating magnetic field (AMF) this nanocarrier can transform electromagnetic energy in heat (above 42 °C) through Néel and Brown relaxation, which makes it applicable for hyperthermia treatment. However, the low chemical and physical stability of MNPs makes their coating necessary. Thus, lipid-based nanoparticles, especially liposomes, have been used to encapsulate MNPs to improve their stability and enable their use as a cancer treatment. This review addresses the main features that make MNPs applicable for treating cancer and the most recent research in the nanomedicine field using hybrid magnetic lipid-based nanoparticles for this purpose.

## 1. Introduction

Cancer is a global health problem, especially for developing countries that shoulder the main burden of disease [1]. According to the International Agency for Research on Cancer (IARC), it is estimated that there were 19.3 million new cancer cases and 10 million deaths worldwide in 2020 [2,3,4]. The study by Sung and coworkers [2] projected 28.4 million new cancer cases by 2040 (a 47% increase over 2020 data), with high numbers of cases in low and medium Human Development Index countries. This projection may undergo significant growth due to the increased prevalence of risk factors associated with the disease [2].

Despite the worldwide scope of cancer, there are significant regional differences in its survival, morbidity, and mortality rates. According to IARC data, the region with the highest number of new cases in 2020 was Asia (58.3%), followed by Europe (22.8%) and the American continents (20.9%). Regarding the types of cancer, female breast cancer is currently the most prevalent type of cancer, followed by lung and prostate cancer [2,5,6]. This could be due to a variety of factors including demographics, lifestyle, genetics, and environmental factors, highlighting that the correlation of many of these factors remains elusive [5,6,7,8].

Traditional cancer treatments, including chemotherapy, radiotherapy, surgery, and targeted therapy, can stabilize or even reduce cancer morbidity and mortality [9]. While cancer treatments are constantly evolving, traditional approaches are costly, have harmful side effects, and can be ineffective due to factors such as metastasis, recurrence, cancer heterogeneity, and distinct genetic profiles, as well as mechanisms of resistance to chemotherapy and radiotherapy. [4,10,11,12]. Factors such as metastasis, recurrence, cancer heterogeneity, and mechanisms of resistance to chemotherapy and radiotherapy are the most frequent causes of therapeutic failure against the disease [11,12]. Faced with therapeutic challenges, new alternative cancer treatments are under development, aiming at new drug delivery systems supported by nanotechnology, with lower toxicity and greater efficiency [4,11].

Nano-scale drug delivery systems are advantageous as they improve drug solubility properties, and chemical stability, in addition to allowing a targeting mechanism for antineoplastic drugs [13]. Passive targeting is known as the high concentration of nanoparticles (NPs) at tumor sites due to the phenomenon known as the enhanced permeability and retention (EPR) effect [12,14]. Active targeting occurs through the specific molecular interaction between NPs and tumor cells or tissues [12,14].

Among the nanosystems currently available, magnetic nanoparticles (MNPs) are a group of inorganic nanosystems that can be composed of pure metals (e.g., Fe, Co, Ni, and Ti), metal oxides (e.g., Fe_3_O_4_ and γ-Fe_2_O_3_), ferrites (BaFe_12_O_19_ and CoFe_2_O_4_), and magnetic nanocomposites [1]. The applicability of these nanosystems in the biomedical field is related to their toxicity and biocompatibility. In general, the key issues affecting these features are the component that is magnetically reactive, the size, shape, and their coating. Some nanoparticles, such as nickel and cobalt nanoparticles, are susceptible to oxidation and toxicity, which makes them difficult to be used for biomedical applications. By contrast, iron oxide nanoparticles have been the most used for biomedical applications due to their low toxicity, excellent magnetic properties, and biocompatibility, which can be reached by adjusting their size and shape [15,16]. Furthermore, the component of MNPs can influence their biodegradability. Iron oxide nanoparticles also have this advantage, given that Fe ions can be reused by cells via normal biochemical pathways [17].

These materials in the nanoscale have unique physical, chemical, and biological features determined by their distinct magnetic properties that are not observed in the bulk material, which make them applicable for diagnosis, therapy, and theragnostics [18]. Due to their great potential, the Food and Drug Administration (FDA) approved them (e.g., Radiogardase^®^, Ferumoxytol^®^, Lumiren^®^, Feraheme^®^, and Endorem^®^) for iron treatment deficiency, as a contrast agent in magnetic resonance imaging (MRI) for cancer detection and monitoring, as a carrier for delivering drugs to specific parts of the human body, and for treating hyperthermia [19,20,21].

However, the physical and chemical stability of MNPs is still a challenge, which makes their coating necessary. In this context, the encapsulation of MNPs in lipid-based nanosystems can increase their stability in the biological environment, favor their therapeutic action against cancer, reduce their toxicity, and increase their biocompatibility [16,22,23,24]. The present review focuses on the most recent research that employed hybrid systems composed of magnetic lipid-based nanoparticles, highlighting their application in cancer therapy.

## 2. Magnetic Nanoparticles’ Properties and Applications

### 2.1. Magnetic Nanoparticles Classification

Materials can be generally classified according to their response to the external applied magnetic field. The orientation of the magnetic moments is important to identify the five basic types of magnetism found in nature: diamagnetism, paramagnetism, ferromagnetism, antiferromagnetism, and ferrimagnetism. Diamagnetic materials are those whose atomic loops generated by the orbital motion of electrons respond in the opposite direction when exposed to an externally applied magnetic field, showing a very weak magnetism. Under the same circumstances, paramagnetic materials are weakly attracted in the same direction as the external magnetic field. However, after the remotion of the magnetic field, the magnetization is null for both. By contrast, ferromagnetic materials (e.g., Fe, Ni, and Co) have aligned atomic magnetic moments even in the absence of an externally applied magnetic field. These materials are highly attracted in the presence of a magnetic field. Nonetheless, above a certain temperature, well known as Currie’s temperature (T_C_), the ferromagnetic substances lose their ferromagnetism and become paramagnetic. By contrast, above a certain temperature, known as Neel’s temperature (T_N_), antiferromagnetic materials (e.g., Mn and Cr) show an antiparallel spin alignment and become paramagnetic. Ferrimagnetic material has populations of atoms with opposite magnetic moments but in an unequal way. Thus, spontaneous magnetization remains. Ferromagnetic and ferrimagnetic materials become paramagnetic when above the T_C_, while antiferromagnetic materials became paramagnetic at temperatures above T_N_ [25,26,27].

**Figure 1:**
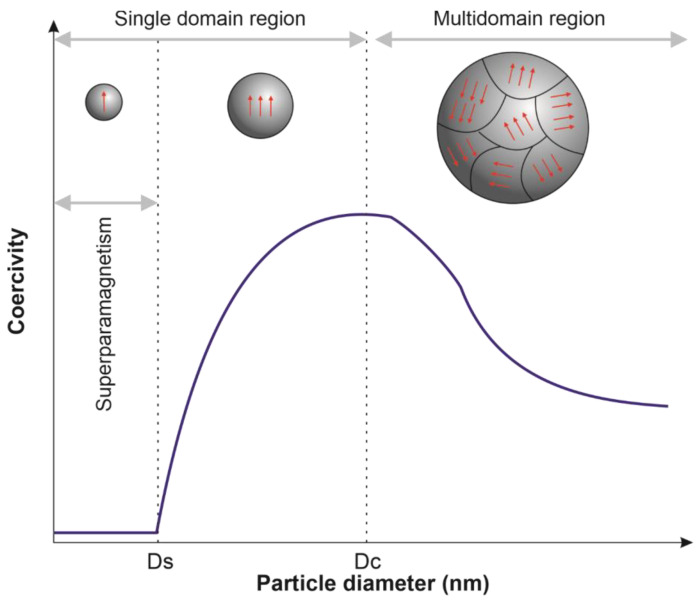
Illustration of the magnetic nanoparticles transition from superparamagnetic to multidomain region according to particle diameter. The red arrows illustrate the orientation of the domains.

**Figure 2:**
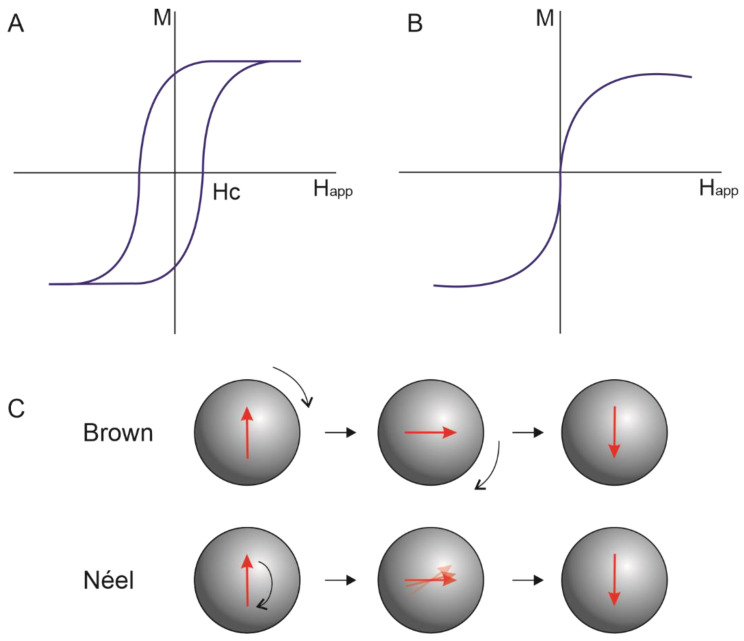
The magnetization curve of (**A**): multidomain structure and (**B**): superparamagnetic nanoparticles. (**C**): Néel and Brown relaxation of magnetic nanoparticles.

Ferromagnetic and ferrimagnetic materials under T_C_ contain regions called domains, in which is observed a mutual alignment in the same direction of all magnetic dipole moments. For particles with larger sizes, multidomain structures formed by different domains separated by domain walls are observed, in which there is a gradual change in the magnetization direction (Figure 1). In the magnetization curve (magnetization (M) vs. magnetic field strength (H_app_)), a slow increase in magnetization induction is initially observed; then, a favorably oriented domain begins to grow, and magnetization induction increases rapidly until reaching a saturation stage. The material remains magnetized after removing the magnetic field because the large aligned domains do not easily return to their initial orientation. An opposite magnetic field can be applied to cancel the magnetic induction. For this, the magnitude of the applied magnetic field must be equal to coercive force (H_C_). When a material is subjected to magnetization in one direction and then the other, a hysteresis loop is observed (Figure 2A) [27].

The presence of multidomains in particles with a size below a critical value (Dc) is energetically unstable, so, in these particles, a single domain is observed. In the single-domain, the coercivity of MNPs gradually decreases with the reduction in particle diameter, and at a second critical value of diameter (D_S_), MNPs are in a superparamagnetic state (Figure 1). The particle diameter in which superparamagnetic behavior appears (D_S_) depends on the nanoparticles’ composition [20,28,29]. In the superparamagnetic state, MNPs exhibit zero coercivity and zero hysteresis (Figure 2B), showing high magnetic susceptibility, fast response to an external magnetic field, and loss of magnetization after the remotion of the magnetic field [15,16,26]. The magnetic properties of MNPs in a superparamagnetic state are the most attractive for biomedical applications, especially for cancer treatment. Among them, the superparamagnetic iron oxide nanoparticles (SPION) have been the most used for hyperthermia, drug delivery, heat-activated drug release, and targeting [16].

### 2.2. Magnetic Nanoparticles’ Application in Cancer Therapy

Magnetic hyperthermia is a therapeutic method that has been used for cancer treatment since 1957. It is based on the capacity of MNPs to transform electromagnetic energy generated by an external alternating magnetic field (AMF) to heat. The increase of tumor microenvironment temperature above 42 °C impacts cancer cell physiology and promotes cell death by initiating a series of pro-apoptotic and apoptotic signaling pathways. Irreversible cellular damage occurs when the external magnetic field generates the heating of MNPs above 46 °C, a process called thermoablation [16,19]. The use of magnetic hyperthermia has been investigated in clinical trials for the treatment of prostate cancer (NCT02033447 and NCT05010759) and osteosarcoma (NCT04316091), but further studies are necessary to understand their effect on other tumors better.

The heating potential of MNPs is influenced by their composition, their concentration, and size [18]. The mechanism in which MNPs with a diameter above Dc under AMF generate heat is mainly governed by losses due to the hysteresis loop as a consequence of the irreversible magnetization process. The heating generated by this mechanism is directly proportional to the area of the hysteresis loop. In MNPs with sizes below Dc, such as SPION, the heating properties are generated by rotating moments. These mechanisms are called Néel and Brownian relaxation (Figure 2C), in which Néel relaxation is caused by the repeated alignments of magnetic spins, while Brownian relaxation occurs owing to rotational diffusion of the whole MNP when they are suspended in a liquid [30,31]. The heat loss of MNPs under AMF will be dependent on the magnetic field frequency, magnetic field strength, and the properties of the MNPs (e.g., particle size, composition, solvent viscosity, and magnetic anisotropy energy constant) [16]. 

**Figure 3:**
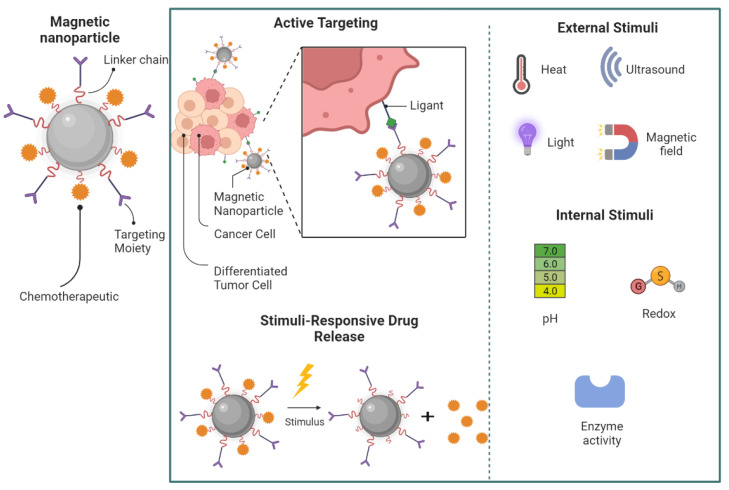
Illustration of active targeting of MNPs and the stimulus that can influence the heat-activated drug release.

The effective magnetic hyperthermia by MNPs depends on several parameters, including the targeting of MNPs, their clearance, and heating power [32]. The targeting by MNPs of tumor cells can occur by passive targeting (i.e., nanoparticles accumulate in the tumor microenvironment as a response to their low particle size, a phenomenon known as the enhanced permeability and retention (EPR) effect), active targeting (i.e., nanoparticles’ internalization into tumor cells due to the specific recognition of ligands on MNPs surface with receptors on tumor cells) (Figure 3), magnetic targeting (i.e., MNPs guided to the tumor microenvironment through an application of an external magnetic field), which can enhance MNPs’ targeting with a subsequent improvement in the cancer cell killing rate [20,33]. Despite the importance of low particle size to promote MNPs’ targeting, nanoparticles below 10 nm can be rapidly eliminated through reticuloendothelial circulation, reducing their concentration in the tumor site [32,34]. The heating power of MNPs, which is their ability to transform electromagnetic energy into heat at a specific amplitude and frequency of AMF, can be estimated by the specific loss power (SLP) or specific absorption rate (SAR). Hence, extensive efforts have been made to produce magnetic nanomaterials possessing high SLP or SAR values to minimize the thermal dose required for sufficient heat and subsequently reduce the risk of side effects [28,32]. The variation in SLP and SAR values can be attributed to several factors, including sample size, concentrations, coating, and magnitude and frequency of the applied field, which should be studied and optimized during their development [32,35,36]. Despite the importance of correctly selecting the AMF amplitude and frequency to obtain high SLP/SAR, for biomedical applications, this selection is essential to avoid undesirable side effects by tissue overheating [37]. Importantly, for human exposure, it is pivotal to maintain the product of the magnetic field strength (H) and its frequency (f) below a threshold safety value known as the Brezovich criterion. In the Brezovich criterion, considering the safety and patient tolerance limits, the product of the frequency and the field amplitude (C = H × f) should remain below 4.85 × 10^8^ Am^−1^s^−1^ [38]. However, in practice, less rigid criteria (C below 5 × 10^9^ Am^−1^s^−1^) is acceptable when hyperthermia is applied in a small body region [39,40].

Besides the potential use of MNPs for hyperthermia treatment, the heating property of these nanosystems can also be explored to promote heat-activated drug release in thermosensitive nanosystems, providing a means to control the drug release in the target tissue (Figure 3) [18,37]. This strategy was used by Hu and coworkers to enhance the release of paclitaxel and doxorubicin from polymer nanocapsules containing MNPs. The release of both drugs significantly increased when an external magnetic field was applied [41]. A similar strategy was also used by Nitica and coworkers to improve the release of doxorubicin from thermosensitive magnetoliposomes for in vitro chemotherapeutic effect [42]. Despite the heat-activated drug release, other stimuli can also influence the drug release, including internal (e.g., pH, redox, hypoxia, and enzymes) and external stimuli (e.g., light irradiation, temperature, and ultrasound) [43]. Other advantages of using magnetic nanoparticles for cancer treatment are that they can be guided specifically to the tumor microenvironment through an application of an external magnetic field and the higher sensitivity of cancer cells to hyperthermia compared with healthy cells [20,44].

Another interesting application of MNPs is for the simultaneous diagnosis and treatment of cancers, which is commonly called theranostics. MNPs can act as a theranostic nanovector, being able to diagnose tumors by Magnetic Resonance Imaging (MRI), deliver drugs to the tumor microenvironment, and monitor the role treatment process by MRI [45,46]. Despite the interesting use of MNPs in theranostics, there is no clinical trial in progress for this application, only for its use in diagnostic (NCT01815333, NCT00920023, NCT00147238, and NCT04682847).

Satpathy and coworkers developed iron oxide nanoparticles coated with a polymer and human epidermal growth factor receptor 2 (HER2) for theranostic application. To produce theranostic nanoparticles, the authors used cisplatin as an antitumoral agent and optical images using near-infrared (NIR) 830 dye, bioluminescence images and MRI. The developed formulation was able to specifically reach the tumor cells with a high level of expression of HER2 during in vivo studies using an orthotopic human ovarian cancer xenograft model, reducing significantly the growth of primary tumor and metastasis. The MRI enables to detection of this response in mice, in addition to helping to identify the lowest antitumoral effect in tumor cells with low expression of HER2 when MRI images were analyzed together with bioluminescence images [47]. 

Galactomannan-loaded SPION functionalized with folate was also used as a theranostic agent for cancer with high expression of folate receptors. This nanosystem significantly inhibited the tumor growth in Ehrlich ascites carcinoma-bearing solid tumor mice in addition to acting as remarkable contrast in MRI, indicating that theranostics is an interesting strategy to treat and simultaneously visualize tumor progress [48].

Despite the great potential of MNPs for cancer treatment, these nanosystems have some drawbacks and challenges to being used clinically. MNPs have the tendency to aggregate and degrade when produced without surface coating [28,49]. In this context, several surface modifications have been proposed to avoid MNPs aggregation and favor their biocompatibility [18,26,28,50]. Among them, lipid-based nanoparticles have been explored in the last years (Table 1), such as liposomes, solid lipid nanoparticles, nanostructured lipid carriers, nanoemulsions, and microemulsions) [23,51,52,53,54]. These nanosystems are generally biocompatible, biodegradable, and able to encapsulate high amounts of hydrophobic drugs for cancer therapy [55,56].

## 3. Techniques for Fabrication of Hybrid Lipid-Magnetic Nanoparticles

The development of hybrid nanosystems containing magnetic nanoparticles requires the initial synthesis of these nanoparticles for subsequent combination with lipid nanosystems. The synthesis of magnetic nanoparticles can be performed by physical, chemical and biological methods. Among the physical methods, the most usual approaches are: microwave irradiation, sonochemical, ultraviolet radiation, laser ablation, thermal decomposition (thermolytic), photochemical, or radical induced. Meanwhile, the chemical method approaches include: supercritical fluid, coprecipitation, use of inorganic matrix as support, and organic solvents, whereas the biological method includes the use of algae, bacteria, fungi and plants as precursors or reactor sources [67,68,69]. 

There are several methods for the preparation of magnetic nanoparticles, each with its own peculiarities regarding the preparation process and the characteristics of the nanoparticles obtained (e.g., size, morphology, stability, and biocompatibility). In general, bottom-up methods result in nanoparticles of various shapes and sizes, while top-down methods result in smaller and well dispersed nanoparticles. Chemical methods consist of different bottom-up approaches, while physical methods cover both approaches. Methods based on biological approaches are efficient, environmentally friendly, and produce biocompatible nanoparticles [70,71].The advantages and disadvantages of some of the main approaches for producing magnetic nanoparticles are discussed in Table 2.

The efficient incorporation of the nanoparticles and the stability of the hybrid system will depend on the intrinsic properties of the nanoparticles, the method of preparation of the lipid system, and the mode of incorporation into the lipid system-incorporated into the lipid phase, incorporated into the aqueous phase, or surface-associated. For example, large hydrophobic nanoparticles incorporated into the liposome bilayer can destabilize the hybrid system [71,72]. The methods for preparing the hybrid system will be according to one of several methods for preparing lipid nanosystems. Every method has its own peculiarities, leading to the formation of systems with different properties. Regardless of the method chosen one must consider the aspects that affect the critical parameters to obtain efficient hybrid systems [73]. Therefore, a planning phase is necessary to obtain hybrid systems with the desirable characteristics, and the reduction of unexpected failures.

**Table 2:** Advantages and limitations of different MNPs preparation techniques.

Technique	Method	Advantages	Limitations	Ref.
Coprecipitation	Chemical	Monodisperse nanoparticles; less harmful materials and processes; easy to execute; high yield, cost-effective.	Critical process factors (pH, metal ions, nature of salt, reaction temperature) influence particle characteristics; difficult to control the shape of nanoparticles.	[74]
Thermal Decomposition	Chemical	Large-scale production of nanoparticles, monodisperse, size and shape controllable, synthesis of smaller nanoparticles, cost-effective.	Production of toxic soluble organic solvents, excessive purification can cause agglomeration of nanoparticles	[75]
Sol-Gel	Chemical	Production in large quantities, controlled size and shape, low cost.	Prolonged reaction time, use of toxic organic solvents, likelihood of contamination of the reactions with by-products.	[76]
Microemulsion	Chemical	Aqueous medium, easy preparation (one-step), monodisperse nanoparticles.	Low-yield synthesis, shape and size depend on the type of surfactant.	[77,78]
Hydrothermal or Solvothermal	Chemical	Monodisperse nanoparticles, production in aqueous media.	Shape and size time-dependent on process pressure and temperature, high cost (high temperature and pressure demand special equipment).	[79]
Mechanical Method	Physical	Fast, inexpensive methods.	Particles with wide size distribution, and product contamination.	[80]
Laser ablation	Physical	Low cost-effective, no toxic residue, easy to apply, monodisperse nanoparticles.	Multiple steps, mechanisms involved in nucleation, phase transition and growth of nanocrystals after laser ablation in liquids are not well understood.	[81]
Wire Explosion	Physical	Safe and clean process, one-step and highly productive process.	Polydisperse nanoparticles	[82]
Biological Methods	Biological	Efficient, clean process, ecofriendly	Polydisperse nanoparticles	[83]

## 4. Hybrid Lipid-Magnetic Nanoparticles

### 4.1. Magnetoliposomes

Liposomes are the first nanoparticle-based formulation with clinical applications, including anticancer therapy (e.g., Doxil^®^, Mepact^®^, DaunoXome^®^, and others) [84]. Liposomes are vesicles that self-assemble into one or more concentric lipid layers in an aqueous medium. They can be composed of natural or synthetic lipids in neutral, cationic, anionic forms, or a combination of them. Liposomes can load hydrophilic compounds in the aqueous core and/or lipophilic compounds in the lipid bilayer due to their vesicular structure. They are capable of improving the biopharmaceutical properties, stability, and toxicity of trapped molecules, with the advantage of being biocompatible and biodegradable. They are also easy to manufacture and can be produced with surface modification or stimulus response (e.g., pH, temperature, oxidative stress) to improve their biological properties [85,86]. However, liposomes can exhibit uncontrolled and slow drug release that can contribute to decreased efficacy of anticancer therapies. Some strategies are explored to ensure drug release within therapeutic ranges over time and overcome this drawback [37,85].

**Figure 4:**
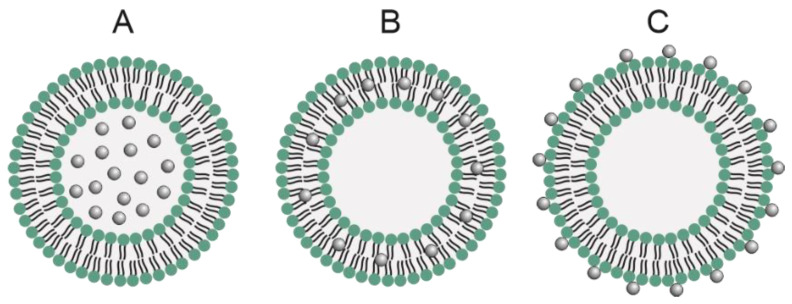
Illustration of magnetoliposomes with magnetic nanoparticles in (**A**): the aqueous lumen of the liposome, (**B**): on the lipid bilayer membrane, and (**C**): bonded to the liposome’s surface.

Magnetoliposomes are hybrid systems that combine liposomes and magnetic nanoparticles, especially SPIONs. These nanoparticles can be attached to liposomes in three ways according to the surface properties and the manufacturing process of the MNPs, which are: in the aqueous lumen of the liposome, on the lipid bilayer membrane, and bonded to the liposome’s surface (Figure 4) [18,37,87]. It was first developed in 1988 by De Cuyper and Joniau [88], and their first application was the controlled release of drugs encapsulated in dipalmitoyl-phosphatidylcholine (DPPC) liposomes in response to heat generated by magnetic hyperthermia [89]. The type of phospholipid in the liposomes is important due to the different lipid transition temperatures (T_m_). DPPC magnetoliposomes (T_m_ 41 ºC) exhibit a faster response to drug release than distearoylphosphatidylcholine (DSPC) magnetoliposomes (T_m_ 55 °C), since less heat generation from the magnetic nanoparticles is required for liposomal membrane destabilization [87]. The liposomal composition must be considered given that it influences the parameters and response of the external stimulus and, therefore, the efficacy of drug delivery and release into the tumor tissue [18,37,87].

These systems have attracted great interest for combining therapeutic and diagnostic applications due to their magnetic, biological, and drug delivery properties. Since 1988, different types of magnetoliposomes have been developed for controlled drug release using the oscillating SPIONs under AMF. Ribeiro and coworkers demonstrated that magnetoliposomes loaded with SPIONs, paclitaxel, and gemcitabine improved gemcitabine entrapment, increased antitumor activity, and provided a controlled release [51]. The SPIONs (8 nm) were produced by coprecipitation method and modified with citric acid for entrapment in the aqueous core of liposomes (DPPC: cholesterol) with a size less than 100 nm. The systems prepared by thin-film hydration and sonication exhibited high encapsulation efficiency for the SPIONs (84%), gemcitabine (57%), and paclitaxel (68%), but especially for gemcitabine in contrast to other liposomes containing gentamicin [90,91]. It is probably due to the association with the SPIONs via citric acid, which decreases the permeability of the drug to the liposomal membranes. At physiological temperature (37 °C), magnetoliposomes exhibited lower than 10% of drug release after 72 h of incubation, while a 30 min application of AMF induced an increase in the release of up to 94% and 42% of gemcitabine and paclitaxel, respectively. In vitro assays showed that combination therapy with hyperthermia was more effective against breast cancer cells (MGSO-3) (27% cell viability) than gemcitabine-paclitaxel therapy (60% cell viability) and hyperthermia alone (50% cell viability) [51]. 

Magnetoliposomes with metallic nanoparticles attached to the aqueous core are the most usual because there is a limitation on the thickness of the lipid bilayer. Therefore, the insertion of metal nanoparticles into the membrane is a challenge compared to insertion into the aqueous core [18,57]. Choi and coworkers produced oleic acid-coated magnetic nanoparticles (~6 nm) via thermal decomposition and inserted them into the lipid bilayer (~3.4 nm thick) of liposomes (DPPC: Dioleoyl-3-trimethylammonium propane). Magnetoliposomes were produced using a solvent-guided method (chloroform) to increase the insertion efficiency of the nanoparticles into the lipid bilayer. The magnetoliposomes were modified with an anti-HER2 antibody or with folate for active targeting of SK-Br3 (HER2-positive) and Hela (FRα-positive) cancer cells. It was used for the separation of the cancer cells by magnetism. The method of entrainment of the metal nanoparticles to the lipid membrane using chloroform proved effective by changing the coloration of the hydration buffer from red to yellowish when compared to the traditional method of hydrating the lipid film. After the incorporation of the nanoparticles into the liposomes (94 nm) they remained stable. An isolation efficiency of 75% was obtained for SK-Br3 due to the targeting of antibodies of the HER2 receptor on the cell surface compared to a 9% recovery efficiency for HeLa cells. High separation efficiency was found for Hela cells when the magnetoliposomes were modified with folate. Confocal microscopy results confirm the presence of the differentially functionalized magnetoliposomes on the surface of cells with the expression of the specific receptors. Antibody-conjugated magnetoliposomes delivered ATTO590 oligonucleotide efficiently into the nucleus of SKBr3 cells. Despite the ability to encapsulate larger nanoparticles, the entrapment efficiency might have been evaluated and compared in both methods since the efficiency of the generated magnetic field is influenced by the size and number of encapsulated nanoparticles. The authors demonstrated by their work that modifications for active targeting in combination with the properties of the magnetoliposomes may be used in association with the control of cellular uptake by magnetic guidance [57]. 

Wang and coworkers used magnetoliposomes to improve the antitumoral efficacy and decrease the adverse effects of 7-(Allylamino)-17-demethoxygeldanamycin (17-AAG)—a heat-shock protein (HSP90) inhibitor that increases the sensitivity of tumor cells to hyperthermia by overcoming thermotolerance. The SPIONs produced by the coprecipitation technique were trapped in liposomes (DPPC: cholesterol) modified with folate. The liposomes (140 nm) showed 87% encapsulation of 17-AAG, and a 57% release rate in 48 h at 43 °C (above the T_m_ of DPPC) compared to 24.3% release in 48 h at 37 °C, and 50% at 55 °C in only 2 h, being temperature-responsive. The targeted magnetoliposomes showed higher inhibition efficiency in SKOV-3 (FRα-positive) than in MCF-7 (FRα-negative) cells, showing the targeting efficiency. The inhibition rate after hyperthermia treatment of the targeted magnetoliposomes in SKOV3 cells was 72% in 24 h compared to 24% for 17-AAG. The apoptosis rate of the magnetoliposomes was 72.6% compared to 33.9% for 17-AAG, consistent with the results of increased levels of the apoptosis-promoting gene Bax, Bcl-xL, and STAT3 mRNA. The targeted magnetoliposomes were able to inhibit tumor growth in a subcutaneous SKOV-3 mouse model by 91.68% and increase survival to 57.8 days. Higher rates than the 57.69% of tumor inhibition and 23.6 days of the 17-AGG treated group. There was a synergistic therapeutic effect with the use of 17-AGG-loaded magnetoliposomes and hyperthermia allowing for new alternatives for the treatment of ovarian cancer [58].

Another strategy employing magnetic nanoparticles for controlled release is the photothermal effect induced by near-infrared (NIR) spectroscopy. Shen and coworkers synthesized hydrophobic SPIONs (~6 nm) by thermal decomposition and trapped them in DPPC and cholesterol liposomes (159 nm) produced by the reverse evaporation method. Doxorubicin loaded into the liposomes via the ammonium sulfate gradient showed 90% encapsulation. After single photothermal irradiation with NIR laser (800 nm, 2 W.cm^−2^, 5 min) the release rate of doxorubicin was 41.6% in 12 h. After two times (0 h and 6 h) of single irradiation, the release rate increased to 66.7% in 12 h, while magnetoliposomes without irradiation released only 10% doxorubicin in 12 h. Confocal microscopy results in MCF-7 cells demonstrated doxorubicin release after irradiation. The viability assay in MCF-7 cells showed that irradiation has no significant influence when the photothermal effect generated is below the T_m_ of DPPC. Mice bearing S180 tumor presented a high intratumoral accumulation of magnetoliposomes 48 h after injection. After 24 h, the magnetic signal intensity of the nanoparticles decreased by 59%, and on magnetic resonance imaging (MRI) evaluation the tumor area became dark. After 24 h of treatment and laser irradiation (5 min), the temperature on the surface of the tumor was 48.6 °C, and after 12 days the tumor had almost disappeared. The results indicate that in vivo photothermal therapy must be performed between 24 h and 48 h after intravenous injection and showed an excellent combined therapeutic and diagnostic effect [59].

Guo and coworkers also produced magnetoliposomes for doxorubicin delivery and release and diagnostic evaluation in neuroblastoma cells (SH-SY5Y cell lines). Liposomes were produced by the lipid film hydration technique, and carboxymethyl dextran (CMD) was used to coat the magnetoliposomes as an alternative to PEGylation due to sensitivity to low pH values. SPIONs (~5 nm) were produced via coprecipitation. The magnetoliposomes (220 nm) were able to encapsulate 96.9% doxorubicin via ammonium gradient. After exposure to low frequency alternating magnetic field (LF-AMF) (45 mT for 30 min), 74% of DOX was released at pH 5.0 within 24 h, while only 35% was released in the absence of LF-AMF. In contrast, at pH 7.0 less than 30% doxorubicin was released in 72 h due to CMD stability. In the absence of LF-AMF and the concentration range of 1 to 5 µg/mL up to 24 h magnetoliposomes showed similar cytotoxicity to free DOX. Therefore, the system was able to decrease the cytotoxicity and increase the efficacy of doxorubicin under LF-AMF. In addition, the magnetoliposomes were shown to be efficient as an MRI agent [60].

Magnetoliposomes are promising systems that combine the properties of two distinct nanosystems. They improve the therapeutic properties and overcome the individual limitations of the isolated nanosystems. There are some papers in the literature involving magnetoliposomes with applications in different areas, including anticancer therapy. However, most work focuses on the physicochemical and colloidal properties of magnetic nanoparticles and magnetoliposomes, and few works focus on studies of the activities of these systems. In view of this, it would be interesting to develop magnetoliposomes and study their biological properties in vitro and in vivo for anticancer therapy. In order to increase the knowledge about the potential of biological activities in preclinical tests, extending the possibility of translation to clinical phase. Since anticancer therapy, despite technological advances, is still a challenge due to different variables such as: variability of the tumor microenvironment, patient compliance, therapeutic resistance and treatment monitoring. In this scenario, therapeutic alternatives with several functions, such as those presented by magnetoliposomes, must be appreciated in order to establish mutual directions in anticancer therapy.

### 4.2. Magnetic Solid Lipid Nanoparticles and Magnetic Nanostructured Lipid Carrier

It is well known that lipid-based nanoparticles are les toxic and biocompatible than inorganic or polymeric nanoparticles. In particular, solid lipid nanoparticles (SLN) have emerged as an alternative to the use of liposomes. SLN are colloidal particles made of a lipid matrix solid at physiological temperature dispersed by surfactants. SLN are also stable, easy to prepare, do not need organic solvents, they are reproducible and scalable [92]. The difference between SLN to nanostructured lipid carries (NLC) is the addition of liquid lipids in the formulation, which will decrease the lipid organization and prevent the drug from expelling. The NLC have the same advantages as SLN, however, instead of low encapsulation over time, by the polymorphic transitions, they have high percentages of drug loading maintained in suitable storage conditions [92,93,94].

Both SLN and NLC are versatile nanostructures that have their surface easily modified according to the goal. They can also encapsulate a range of structures to improve their therapeutic function. Thus, SLN and NLC are promising alternatives to coating MNPs, performing high tumor distributions followed by a successful localized magnetic hyperthermia [22,95,96]. In addition, these lipid-based nanoparticles allow the encapsulation of a antitumor drug performing a multifunctional approach and also coating a magnetic core aiming theragnostic [69,97].

Ahmadifard and coworkers developed letrozole-loaded in chitosan-coated magnetic SLN aiming their controlled release in tumoral cells. First, the authors prepared a magnetic nanostructure adopting the chemical co-precipitation method by a mixture of iron salts II (FeCl_2_.4H_2_O) and iron salts III (FeCl_3_.6H_2_O) at 75 °C. The pH increased by the addition of NaOH solution, leading to the formation of the magnetics nanostructures. The authors prepared the magnetic SLN by a combination of methods of solvent evaporation ultrasonic. The SLN were composed of stearic acid and tripalmitin glycerol as lipids and the surfactant was dioctyl sulfosuccinate sodium salt. Finally, the coating with chitosan was made at the end of the process. Several methods of characterization were performed, including the application of magnetic field and antioxidant activity. The encapsulation of letrozole in magnetic SLN (190 nm) was about 90%, which was delivered without and with low-frequency pulsed magnetic fields. The application of this magnetic field enhanced the drug release, with 50% of drug release after 1 h The magnetic fields delayed the drug release by about 20% instead of the same amount being released in 12 h without the application of the magnetic field. The authors showed the capacity of letrozole loaded in chitosan-coated magnetic SLN in the reduction of tumoral cells viabilities and also an antioxidant activity by neutralization of reactive oxygen species (ROS) and reactive nitrogen species (RNS) [63].

Iacobazzi and coworkers worked on the development of SLN co-encapsulating SPIONs and sorafenib. They exploited the hypothesis that this nanosystem can act as magnetic-guided drug delivery to the liver. The SLN were prepared by hot homogenization and loaded both, SPIONs and sorafenib. They were presented in the range of 135–164 nm, increasing with the SPIONs percentage (1, 2, 2.5, and 3%). The authors observed, in vitro, the accumulation of the magnetic formulations inside HepG-2 liver cancer cells, and this accumulation was enhanced in the presence of an external magnetic field. The nanoparticles modulated the release of encapsulated sorafenib in the culture medium in a time-dependent manner. The authors studied the behavior of the nanoparticles in a magnetic field designed to simulate the liver blood flow and designed an under-skin implant. In vivo experiments using under-skin implantation, showed a high Fe content in liver tissue that corroborated with the authors’ hypothesis [61].

Grillone and coworkers also encapsulated sorafenib in magnetic SLN and the authors focused on the liver tissue as well. They studied the application of these nanoparticles for hepatocarcinoma. The magnetic SLN were produced through an oil-in-water homogenization process for encapsulating both SPION (10 nm) and sorafenib. Magnetic SLN were about 250 nm with regular shape confirmed by atomic force microscopy. The images showed a magnetic core involved by lipids and the encapsulated and surface localized SPION was also characterized using electron density by transmission electron microscopy. The magnetic nanoparticles were able to accumulate in the target cells and sorafenib acted in proliferation inhibition [62]. Magnetic SLN loading sorafenib were studied in another work that aims to improve its oral bioavailability and also their liver accumulation, as previously reported by other authors. In vivo studies pointed out magnetic SLN magnetic had accumulated in the liver more significantly than in oral suspension, showing the magnetic capability of bioaccumulation [98].

Another work developed Nutlin-3a and superparamagnetic nanoparticles encapsulated in SLN for glioblastoma multiforme treatment. The magnetic SLN (180 nm) were prepared by solvent evaporation technique. The authors characterized the system by analyzing their physicochemical properties, their effects on U-87 MG glioblastoma cells, and their ability to cross the blood-brain barrier in an in vitro model using bEnd.3 cells. The nanoparticles showed colloidal stability and in vitro ability to cross the blood-brain barrier in vitro and reach the U-87 MG cells. The authors demonstrated that magnetic SLN in the presence of a magnet tend to be internalized in bEnd.3 cells to reach glioma cells, which was not extensively observed in the absence of a magnet. Furthermore, the greater pro-apoptotic activity of this formulation was achieved compared to free Nutlin-3a [99].

Allam and coworkers developed thermoresponsive solid lipid nanoparticles coating a magnetic core aiming to enhance the solubility and stability of camptothecin and perform hyperthermia. SPION was first prepared by co-precipitation method followed by their encapsulation into SLN (<200 nm) composed of DPPC (T_m_ = 41 °C) and dipalmitoylphosphatidylglycerol (DPPG) lipids. The magnetic SLN showed a thermoresponsive drug release due to the lipid T_m_ below the heat temperature reached by SPION under AMF, with the faster release at 45 °C. The authors demonstrated magnetic SLN did not affect the human Jurkat lymphoma cell viability when SPION was stimulated to heat to 40 °C and 42 °C for up to 30 min. However, a significant cell death was observed when SPION promoted heating of 45 °C during 10 min of exposure [23]. 

Magnetic SLN were studied for colon adenocarcinoma [100], cancer in general [101,102,103], and in inflammatory process [104]. The SLN are also used as a contrast agent for magnetic resonance imaging application. The easy accumulation of these nanoparticles in tissue such as kidneys, bones, spleen and brain corroborated their use for this purpose. Their efficient and rapid biodistribution are related to their biocompatibility and this is the main advantage of this system to carry paramagnetics contrast agents [105].

Rodenak-Kladniew and coworkers developed a hybrid magnetic NLC. The nanosystem was composed of myristyl myristate coated with chitosan, in which was incorporated the 1,8-cineole and MNPs. MNPs (20 nm) were synthesized through co-precipitation method, while NLC (~250 nm) was produced by homogenization with the ultra-sonication method. The absence of magnetic hysteresis indicated that the MNPS produced were in a superparamagnetic state. The antitumoral activity of magnetic NLC was evaluated in HepG2 (human hepatoma) and A549 (lung cancer) cell lines, which presented a higher effect in viability in HepG2. Furthermore, no cytotoxicity effect was observed in normal cells lies (WI-38 cells), demonstrating the specificity of the developed formulation. The authors also observed that the uptake rate increase significantly when the coating with chitosan was performed. The magnetization behavior was studied using a vibrating scanning magnetometer and the increment of the temperature from 37 °C to 45 °C increased the release of 1,8-cineole suggesting a possibility to perform a hyperthermia treatment [64].

As observed in several already-mentioned works in this review, the modification of the temperature affects directly the release of the drugs encapsulated in both nanosystems, magnetic SLN and NLC. Some works suggested that this behavior of release is related to the transition temperatures of the lipids used in the formulations. The increment in the temperatures aiming treatments based on hyperthermia affects the lipid coating, making easy the release of encapsulated molecules. Yoozbash and coworkers observed this release behavior for curcumin, in which the increment in the temperature increased the release by almost 20% [106].

Ong and coworkers developed a magnetic NLC through the hot ultrasonication method to perform hyperthermia and deliver methotrexate as a multifunctional treatment for breast cancer. The developed magnetic NLC (214 nm) showed fast internalization in cells, with increased apoptotic-mediated cell death in the presence of AMF. The authors studied the pathways of internalization of this system and determined by inhibitors that magnetic NLC entry in the cells by caveolae-mediated endocytosis in a time-dependent manner [107]. In addition, a magnetic NLC was developed for magnetic resonance imaging of hepatic tumor. For this, the authors used as ferritin reporter genes as a guide. MRI contrast agent for imaging hepatic tumor was achieved with high sensibility generating accurate images [108].

Regarding SLN and NLC, the encapsulation of magnetic nanoparticles seems to be an interesting strategy to improve biocompatibility with tissues as well as increase cell uptake, since most of the lipids used for these formulations are similar to lipids naturally found in cell membranes, cell matrices.

### 4.3. Magnetic Nanoemulsion and Microemulsion

Nanoemulsion and microemulsion are colloidal dispersion systems formed by two immiscible liquids (water and oil) stabilized with a surfactant, which reduces the interfacial tension. The main difference between nano and microemulsions is in relation to stability, nanoemulsions are thermodynamically unstable and kinetically stable, while microemulsions are thermodynamically stable [109]. Among the advantages of both systems, their ability to increase drug stability and solubility in aqueous media and sustain the drug release should be highlighted. Furthermore, they are biocompatible and biodegradable nanosystems [110,111].

De Paula and coworkers developed magnetic nanoemulsions to deliver MNPs and chloroaluminium phthalocyanine for combining hyperthermia and photodynamic therapy on mesenchymal stem cells treatment, respectively. For developing magnetic nanoemulsion (173.6 nm), iron oxide nanoparticles were added into the aqueous phase and chloroaluminium phthalocyanine was added into the oil phase. The magnetic nanoemulsion/chloroaluminium phthalocyanine exposed only to an AMF for hyperthermia reduced about 10% of the cell viability of bone marrow mesenchymal stem cells in vitro. However, when hyperthermia was associated with photodynamic therapy there was an increase in cell death, with cell viability values around 70%. Thus, the synergistic effect of the combination of both techniques was evidenced, being a potential alternative for treatment against other cancer cell lines [65]. Another study conducted by the same research group investigated the effect of phthalocyanine-loaded magnetic nanoemulsion in the treatment of glioblastoma multiform combining hyperthermia and photodynamic therapy. The in vitro studies demonstrated that this magnetic nanoemulsion was able to reduce 70% of the cellular viability of glioblastoma cells when both hyperthermia and photodynamic therapy were applied. In addition, confocal images indicated the cellular uptake of magnetic nanoemulsion in U-87 MG and T98G cells, demonstrating that this formulation has also antitumoral potential for treating glioblastoma multiform [112].

In the same way, Pellose and coworkers combined hyperthermia and photodynamic therapy for breast cancer treatment through the encapsulation of MNPs and chlorin E6 (Chle6), a photosensitizer, into nanoemulsions composed of cholesteryl oleate, phosphatidylcholine, triolein, and cholesterol. The developed magnetic nanoemulsion showed a particle size of 153 nm. In the in vitro cell viability assay against MCF-7 cells, there was no induction of cytotoxicity without the stimulation of an AMF or red light. After the magnetic nanoemulsion exposure to AMF and red light, there was increased cytotoxicity, reducing the cell viability by more than 80%, in addition to targeting overexpressed low-density lipoprotein receptors on breast cancer tumor cells. Furthermore, magnetic nanoemulsions were found to be less susceptible to cytotoxicity after the isolated application of hyperthermia than PDT in MCF-7 tumor cells [53]. 

A thermosensitive magnetic nanoemulsion hydrogel containing Zn ferrite MNPs coated with oleic acid was developed by Wu and coworkers through the sonication emulsification method. The magnetic nanoemulsion had a small hydrodynamic particle size (55 nm) and a high magnetization (98.7 emu/g Fe). Mice bearing 4T1 tumors were treated with the thermosensitive magnetic nanoemulsion hydrogel in the presence of an AMF for magnetic hyperthermia, keeping it located in the center of the tumor. The treatment with hyperthermia significantly reduced the tumor volume, being almost completely eliminated after 3 months of treatment. By contrast, animals treated with magnetic nanoemulsion in the absence of AMF and the control group did not reduce tumor volume, showing an average life span of about 12 to 40 days. In this way, the magnetic field becomes indispensable to promote the targeting and the antitumor effect of the formulation through magnetic hyperthermia [66]. 

Natesan and coworkers produced a magnetic microemulsion loaded with camptothecin, an alkaloid with antitumor action by inhibiting topoisomerase. The MNPs were produced through the co-precipitation method and encapsulated into microemulsion by ultrasonication technique. The developed system showed a particle size of 158 nm and a superparamagnetic state. The in vitro cytotoxicity assay demonstrated that magnetic microemulsion significantly reduced the MCF-7 cell viability (IC_50_ = 129 ± 3.9 ng/mL), with lower IC_50_ values than microemulsion (IC_50_ = 178 ± 34.3 ng/mL). Furthermore, the formulation did not show changes in the DNA of lymphocytes in the genotoxicity assay using the comet model. In the in vivo model of orthotopic breast cancer, the application of an external magnetic field of 1000 gauss for 1 h demonstrated magnetic microemulsion targeting and high accumulation in the breast tumor tissue, unlike the camptothecin-loaded microemulsion without MNPs and magnetic field stimulation. Thus, the presence of MNPs and a magnetic field promoted the active targeting of the magnetic microemulsion to the target tissue, making it possible to have a greater concentration of camptothecin in the tumor tissue to promote the antitumor effect with reduced systemic toxicity [52].

Wang and coworkers developed a magnetic microemulsion functionalized with T7 peptide and AS1411 aptamer for active targeting of shikonin and docetaxel to glioma cells. The authors proposed a triple-glioma targeted delivery through the magnetically guided microemulsion by an external applied magnetic field and the specific recognition of AS1411 aptamer and T7 peptide on microemulsion surface by nucleolin and transferrin receptors overexpressed in glioma cells and the blood-brain barrier, respectively. The MNPs showed a particle size of about 8 nm and a superparamagnetic behavior, while magnetic microemulsion had a particle size of about 35 nm. The combination of all targeting strategies improved magnetic microemulsion uptake into murine glioma cells (G422) and showed the strongest antiproliferative activity compared with other microemulsions that used only one active targeting strategy. Thus, the developed systems were shown to be a promising treatment for glioma due to their synergic activity [113].

In general, we can show that the application of AMF in the addressed magnetic nano and microemulsions improves the cytotoxic and antitumor activity in vivo. However, studies are not limited to just this magnetic aspect, adding different techniques in order to provide enhancement of its actions, such as the association with the photodynamic therapy technique, use of functional agents (peptide and aptamer), and incorporation into a thermosensitive system, to promote better targeting of target cells, which would directly imply the reduction of adverse effects. It demonstrates the potential of these drug delivery systems for cancer therapy.

## 5. Conclusions and Future Perspectives

Nanotechnology has gained special attention in the last decades in the biomedicine field, especially for the treatment of cancers. Among the nanosystems currently available, MNPs have demonstrated great potential due to their ability to specifically reach the tumor tissue, through the external application of a magnetic field in a desirable site, and to transform electromagnetic energy in heat when exposed to an AMF, causing tumor cell death by the hyperthermia process. In addition, this nanosystem allows theranostics, being able to act in both diagnosis and treatment, avoiding extra steps in the treatment schedule for patients. This combination can increase the quality of treatment and accuracy regarding long-term treatments in solid tumors. 

The present review addressed lipid-based nanosystems used to carry MNPs and improve their physical and chemical stability for enabling their use in clinical. Among the lipid-based nanosystems, liposomes have been the most used for delivering MNPs and drugs to tumors, especially the thermosensitive liposomes, which are those liposomes composed of lipids with phase transition temperature above the heating temperature of MNPs. In the thermosensitive liposomes, the heating of MNPs causes changes in the lipid physical state form, which enable a fast drug release when AMF is applied. Lipids with this feature have also been used to produce magnetic NLC for a heat-activated drug release, which has proved to be an interesting strategy to increase drug concentration in the tumor microenvironment.

Moreover, the research studies addressed in this review demonstrated the great potential of combining the delivery of magnetic nanoparticles with other molecules into lipid-based nanosystems, including chemotherapeutic agents (e.g., paclitaxel, docetaxel, doxorubicin, and curcumin) and photosensitizers, thus demonstrating the great in vitro and in vivo antitumoral potential of hyperthermia combined with chemotherapy and photodynamic therapy. Despite the promising application of hybrid magnetic lipid-based nanoparticles in the treatment of various tumors, there are still no clinical trials reporting their effect in humans, possibly due to the recent development of these systems. Furthermore, we believe that in the near future the scientific community will be well acquainted with this delivery system, which has great potential for therapy, diagnostic, and theranostic.

Therefore, we encourage future in vitro and in vivo studies and clinical trials using hybrid magnetic lipid-based nanoparticles in order to improve the knowledge about its potential application in cancer therapy alone or in combination with chemotherapy and photodynamic therapy.

## Figures and Tables

**Table 1:** Recent studies that investigated the antitumoral effect of hybrid magnetic lipid-based nanoparticles.

Nanosystem	Drug	Preparation Method	MNPs Encapsulation Efficiency	Treatment Method	Effect	Ref
Magnetoliposomes	Paclitaxel and gemcitabine	Coprecipitation (MNPs) and thin-film hydration (ML)	84%	Chemotherapy and hyperthermia	Increased gemcitabine encapsulation, 10-fold increase in drug release under AMF, 2.1-fold increase in cytotoxicity on MGSO-3 cells	[51]
Magnetoliposomes	ATTO590 oligonucleotide	Thermal decomposition (MNP) and solvent-guided method (ML)	n.r	Chemotherapy	Size-independent MNP loading increases using the solvent-guided method than the film hydration method. Increased separation efficiency of cancer cells from functionalized systems.	[57]
Magnetoliposomes	17-AAG	Coprecipitation (MNP) and thin-film hydration (ML)	n.r.	Chemotherapy and hyperthermia	Higher inhibition efficiency on SKOV-3 (FRα-positive), increased apoptosis rate and apoptosis-promoting genes, increased survival and tumor inhibition rate in xenograft models.	[58]
Magnetoliposomes	Doxorubicin	Thermal decomposition (MNP) and reverse evaporation (ML)	n.r	Photothermal and chemotherapy	Increased release rate on irradiation, increased accumulation in brain tissue in a mice model, therapeutic and diagnostic MRI synergism.	[59]
Magnetoliposomes	Doxorubicin	Coprecipitation (MNP)and thin-film hydration (ML)	83%	Chemotherapy	Increased release rate on LF-AMF and at acidic pH, decreased cytotoxicity of NPMs, improved efficacy of doxorubicin.	[60]
Superparamagnetic solid lipid nanoparticles	Sorafenib	Microemulsion (MNP) and Oil-in-water homogenization process (SLN)	-	Chemotherapy	Increase in vitro and in vitro accumulation into liver cancer cells.	[61]
Magnetic soli-lipid nanoparticles	Sorafenib	Oil-in-water homogenization	-	Chemotherapy	The developed formulation accumulated into liver tumor cells and inhibited their growth	[62]
Magnetic solid lipid nanoparticles	Letrozole	Coprecipitation (MNPs) and solvent evaporation-ultrasonic (SLN)	-	Chemotherapy	Increased the antitumoral efficiency of letrozole.	[63]
Magnetic Nanostructured lipid carrier	1,8-cineole	Ultra-sonication (NLC)	-	Chemotherapy and hyperthermia	Higher antitumoral effect in tumoral cells than normal ones.	[64]
Nanoemulsion	Chloroaluminum phthalocyanine	Spontaneous emulsification	-	Hyperthermia and photodynamic therapy	Synergism between hyperthermia and PDT techniques in cell death	[65]
Nanoemulsion hydrogel	-	Thermal decomposition (MNP) and emulsification by sonication (nanoemulsion)	-	Hyperthermia	Active targeting and 4T1 tumor reduction in vivo in the presence of an alternating current magnetic field	[66]
Nanoemulsion	Chlorin E6	Emulsification by ultrasonic irradiation	-	Hyperthermia and photodynamic therapy	Increased cytotoxicity with combination of hyperthermia and PDT against MCF-7 cells	[53]
Microemulsion	Camptothecin	Coprecipitation (MNP) and emulsification by ultrasonication (microemulsion)	-	Chemotherapy	Active targeting and greater accumulation of camptothecin to the tumor after magnetic field application	[52]

Legend: 17-AAG: 7-Allylamio-17-desmethoxygeldanamycin; CHO: cholesterol, CMD: carboxymethyl dextran, DPPC: 1,2-dipalmitoyl-sn-glycero-3-phosphocholine, DSPE-PEG_2000_-folato: 1,2-diestearoil-sn-glicero-3-fosfoetanolamina-N-[folato(polietilenoglicol)-2000], ML: magnetoliposomes, MNP: magnetic nanoparticle, mPEG_2000_-DSPE: 1,2-dipalmitoil-sn-glicero-3-fosfoetanolamina-N-[metoxi(polietilenoglicol)-2000], NLC: nanostructured lipid carrier, n.r: not reported, PEG-2-PE: 1,2-dipalmitoyl-sn-glycero-3-phosphoethanolamine-N-[methoxy(polyethyleneglycol)-2000] (ammonium salt), SLN: solid lipid nanoparticles, SPIONs: superparamagnetic iron oxide nanoparticles.

## Data Availability

Not applicable.
